# CoCrMo surface modifications affect biocompatibility, adhesion, and inflammation in human osteoblasts

**DOI:** 10.1038/s41598-020-58742-9

**Published:** 2020-02-03

**Authors:** Birgit Lohberger, Nicole Stuendl, Dietmar Glaenzer, Beate Rinner, Nicholas Donohue, Helga C. Lichtenegger, Leon Ploszczanski, Andreas Leithner

**Affiliations:** 1 Department of Orthopedics and Trauma, Medical University Graz, Graz, Austria; 2 Division of Biomedical Research, Medical University Graz, Graz, Austria; 3 Department of Material Sciences and Process Engineering, Institute of Physics and Materials Science, University of Natural Resources and Life Sciences (BOKU), Vienna, Austria

**Keywords:** Cell growth, Translational research

## Abstract

In this study, different surface modifications were performed on a Cobalt-Chrome-Molybdenum (CoCrMo) alloy and the effects on cell viability and cytotoxicity as well as the adhesion potential of human osteoblasts (hFOB) and their inflammation reaction were investigated *in vitro*. CoCrMo discs were coated with TiN, with polished and porous coated surfaces, or with pure titanum (cpTi) surfaces and examined by Scanning Electron Microscopy to evaluate surface modifications. *In vitro* cell viability, adhesion behaviour, and expression of inflammation markers of hFOB human osteoblasts were measured via CellTiter-Glo, CytoTox, ELISA, and RT-PCR respectively. All results were compared to CoCrMo without surface modifications. The biocompatibility data showed high compatibility for the TiN hard coatings. Likewise, the porous surface coating increased cell viability significantly, compared to an untreated CoCrMo alloy. None of the investigated materials influenced cytotoxicity. Different surface modifications did not influence expression of fibronectin, although TiN, porous surface coatings and polished surfaces showed highly significant reductions in integrin subunit expression. In addition to the regulation of adhesion potential these three surfaces stimulated an anti-inflammatory response by osteocytes. Improved biocompatibility and adhesion properties may contribute to better osteointegration of prosthetics.

## Introduction

Cobalt-Chrome-Molybdenum (CoCrMo) alloys are bioactive materials that display high corrosion resistance and favourable mechanical properties. For these reasons, they are frequently used as implants in orthopedic surgery, especially as replacements for hip and knee joints^1^.

Multiple approaches have been proposed to increase biocompatibility of CoCrMo alloys and stimulate new bone formation by enhancing osteoblast adhesions and proliferation^2–4^.

The most important aspect of a metallic implant is biocompatibility and how it reacts with living cells^5^. Materials identified as biocompatible can be embedded within living tissue without eliciting negative or unwanted effects^6^. Altering the surface of implants can be used to improve their design. Furthermore, improved implant surfaces can promote bone integration under *in vivo* conditions^7^. Interactions between the implant surface and bone tissue can be improved through the following techniques: polishing, acid etching, sand-blasting, plasma spraying as well as applying bioactive coatings^8–10^. Implants with high surface roughness speed up biological fixation, leading to bone healing^11,12^. Rough implant surfaces can be produced by applying titanium particles to the surface of the implant using plasma spraying (TPS)^13,14^. The increased roughness of TPS treated surfaces allows better ingrowth of bone cells and osseointegration. Apart from rough coatings, osseointegration can also be improved using mechanical polishing, as this process creates residual stress and produces a deformed layer on the surface^15^.

Using an *in vitro* model this study examined how cell viability and cytotoxicity, osteoblast adhesion and expression of inflammation markers were affected by polished or porous coated surface modifications of the CoCrMo alloy, respectively titanium nitride (TiN) or pure titanium (cpTi) coating.

## Results

### Surface characteristics

**Figure 1:**
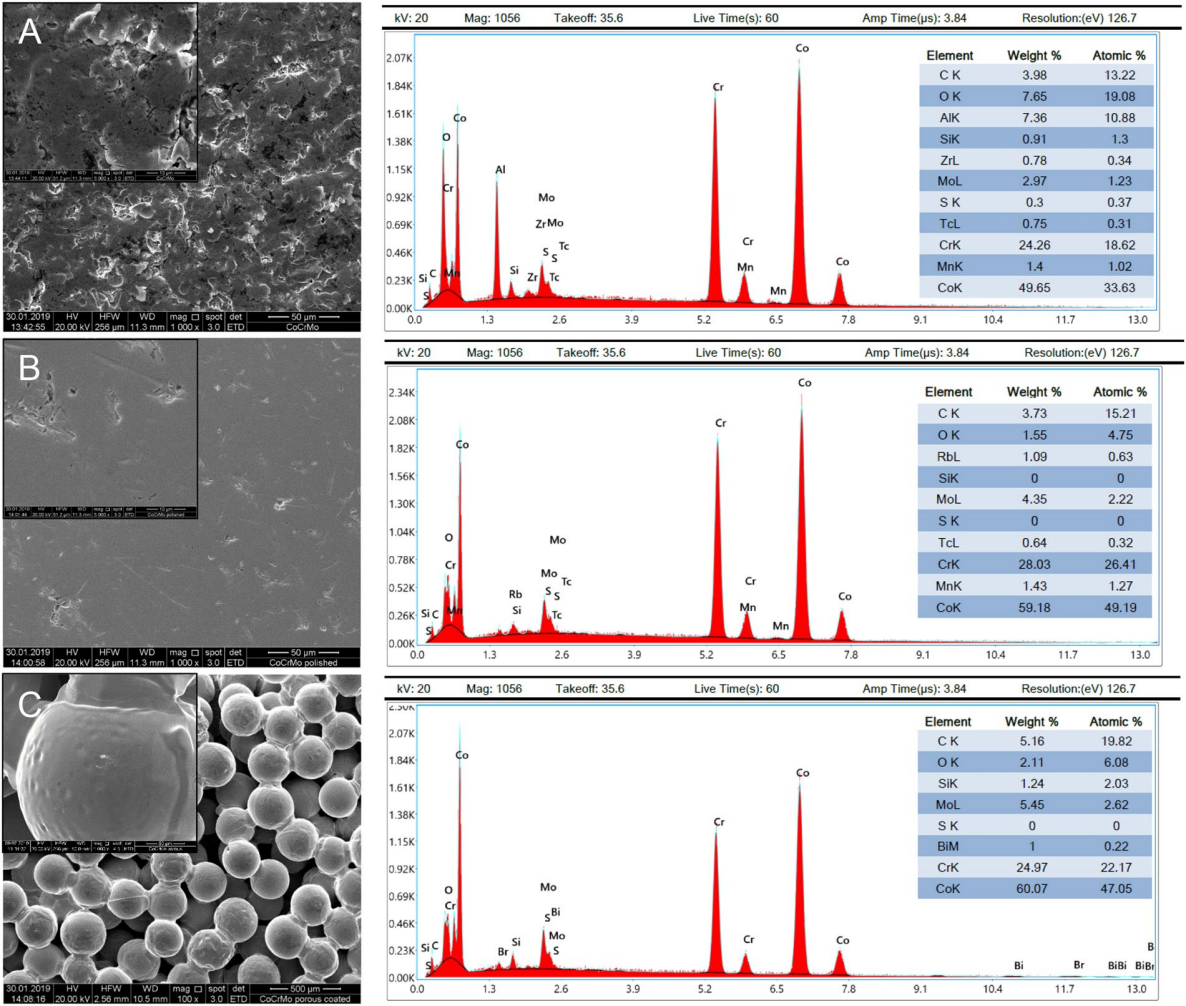
Scanning electron microscopy (SEM) and their corresponding energy-dispersive X-ray (EDX) analysis of CoCrMo alloy showing representative phases and textures. (**A**) CoCrMo 1000x and 5000x magnification, (**B**) CoCrMo polished 1000x and 5000x magnification; and (**C**) CoCrMo porous coated 100x and 1000x magnification.

**Figure 2:**
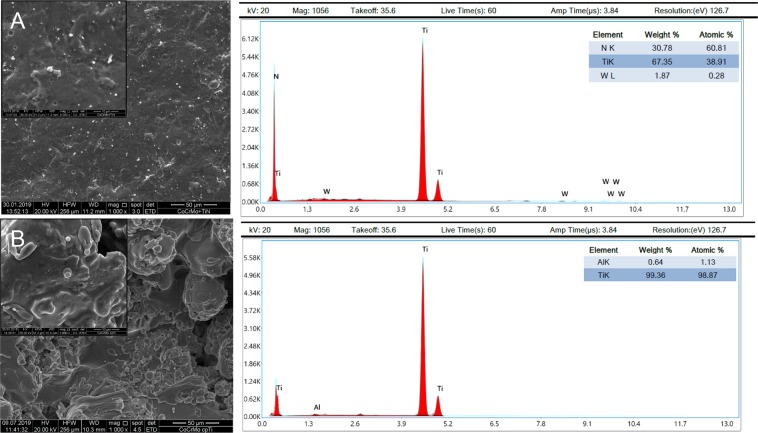
Scanning electron microscopy (SEM) and their corresponding energy-dispersive X-ray (EDX) analysis of (**A**) CoCrMo TiN 1000x and 5000x magnification, and (**B**) CoCrMo cpTi 1000x and 5000x magnification.

Before starting cell culture based analyses, the different surface modifications were characterized by SEM and corresponding EDX. The SEM microscopic images revealed significant differences in morphology of the uncoated CoCrMo discs (Fig. 1A; 1000x and 5000x magnification) and their modifications. As you can see well the polished surface showed very few structures (Fig. 1B). The spherical structures of the porous coated surface can be seen particularly well at 100x and 1000x magnification (Fig. 1C). The spheroid size was between 250–355 µm. EDX analyzis revealed no differences in these three groups regarding the composition of the chemical elements. TiN and cpTi coatings on the other hand, fundamentally changed the surface quality (Fig. 2). Macroscopically, the TiN coating showed a metallic, golden yellow appearance in which the layer bonded extremely well to the implant. The TiN coated surface is slightly roughened (R_a_ < 0.05 µm) (Fig. 2A). The special coating process of the vacuum plasma spraying creates a structured surface with rounded elements when coating with commercially pure titanium (cpTi) (Fig. 2B).

### *In vitro* biocompatibility assays

**Figure 3:**
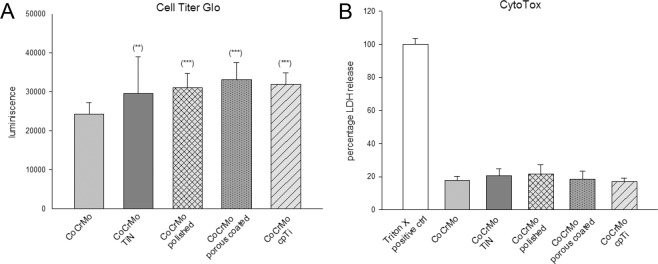
Biocompatibility assays of CoCrMo alloy surface modifications. (**A**) The cell viability of human osteoblasts increased significantly on all surface modifications, especially on TiN coated and porous coated surfaces. (**B**) The cytotixicity assay showed no significant changes (mean ± SEM; N = 9; measured in quadruplicates).

To analyze the biocompatibility of the different surface modifications the CellTiter-Glo^®^ viability and the CytoTox-ONE™ cytotoxicity assays were carried out after four days of incubation. The cells were spread evenly on all surfaces. The original unmodified CoCrMo was used as a control sample (100%) to compare differences to the modified surfaces. Data were shown as mean ± SEM; N = 9; measured in quadruplicates (Fig. 3A). All surfaces showed highly increased osteoblast viability due the different surface modifications. The ceramic TiN coating is characterized by high biocompatibility and showed an enhancement to 145.6 ± 18.4% (***p = 6.2E-14). The polished surface increased viability to 129.4 ± 10.9% (***p = 2.1E-17) and the porous coated surface to 144.1 ± 13.3% (***p = 1.1E-20). Furthermore, the commercially pure titanium (cpTi) improved the viability to 126.7 ± 13.8% (p = 1.8E-13). Lactate dehydrogenase (LDH) is released from cells when membranes are disrupted. In necrotic cells the membrane is disrupted and LDH escapes the cell. The amount of fluorescence is proportional to the number of lysed cells. LDH release and by extension, cytotoxicity remained stable between all groups (Fig. 3B) (mean ± SEM; N = 9, measured in quadruplicates).

### Cellular adhesion proteins on materials

**Figure 4:**
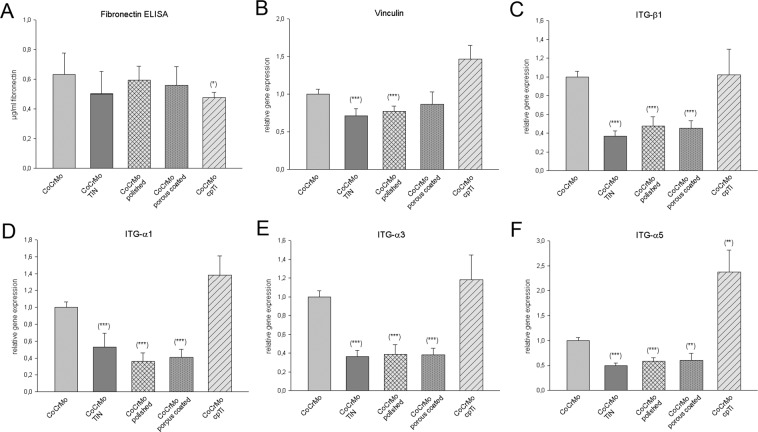
Analysis of the adhesion potential on different CoCrMo surface modifications. (**A**) Compared to the CoCrMo control group, fibronectin expression showed only slight changes. The relative gene expression of (**B**) vinculin and (**C**) the integrin subunits ITG-β1, and **D–F**) ITG-α1, -α3, α5 (mean ± SD; N = 6; measured in triplicates). Expression of all integrin subunits was significantly decreased in TiN coated, polished, and porous coated surfaces and increased in cpTi, compard to the unmodified CoCrMo alloy.

Fibronectin, a glycoprotein of the extracellular matrix, binds to membrane-spanning integrin receptor proteins associated with tissue remodeling and repair. Compared to the CoCrMo control groups (0.63 ± 0.14 µg/ml) fibronectin synthesis was significantly decreased on cpTi surfaces (0.48 ± 0.03; *p = 0.01) (Fig. 4A). No significant differences were detected in the other groups. The membrane-cytoskeleton interactions were analyzed using gene expression analysis of vinculin, which is involved in integrin-mediated cell-matrix adhesion and cadherin-mediated cell-cell junctions. The ceramic TiN coating (relative mRNA levels: 0.71 ± 0.08; ***p = 0.0001) and the polished CoCrMo surface (0.77 ± 0.07; *p = 0.0001) revealed a significant decrease in vinculin expression, whereas the cpTi overlay enhanced the formation of vinculin (Fig. 4B). ITG-α3β1 and ITG-α5β1 play an especially important role as fibronectin receptors in osteoblasts. Therefore, we investigated the expression of the integrin subunits α1, α3, α5, and β1 by real time RT-PCR after four days. The CoCrMo control cells served as the reference value (ratio = 1). All values were mean ± SD of independent experiments performed in triplicate (N = 6). The expression of the integrin subunits showed a very consistent representation. All three α-subunits as well as the β1-subunit showed a significant reduction in expression in the following three groups: CoCrMo TiN, CoCrMo polished, and CoCrMo porous coated. The expression of the integrin subunits of CoCrMo cpTi, on the other hand, is significantly enhanced (Fig. 4C–F). All corresponding values are shown in Table 1.

**Table 1:** The expression levels of the integrin subunits were normalized to the reference genes and the relative expression ratio was expressed as 2^ΔΔCt^. CoCrMo control cells served as the reference value (ratio = 1). All values were mean ± SD of independent experiments performed in triplicate (N = 6).

	ITG-β1	ITG-α1	ITG-α3	ITG-α5
TiN	0.37 ± 0.06 ***p = 6.6E-9	0.53 ± 0.16 ***p = 0.0005	0.36 ± 0.06 ***p = 1.02E-8	0.49 ± 0.05 ***p = 5.2E-8
polished	0.48 ± 0.09 ***p = 1.9E-6	0.36 ± 0.09 ***p = 4.1E-7	0.39 ± 0.09 ***p = 8.4E-7	0.58 ± 0.07 ***p = 8.5E-7
porous coated	0.45 ± 0.07 ***p = 1.5E-6	0.41 ± 0.09 ***p = 8.3E-6	0.38 ± 0.07 ***p = 3.2E-7	0.60 ± 0.13 **p = 0.001
cpTi	0.97 ± 0.33 n.s.	1.38 ± 0.22 *p = 0.03	1.18 ± 0.26 n.s.	2.37 ± 0.44 **p = 0.008

### Induction of inflammation and TLR4 receptor

**Figure 5:**
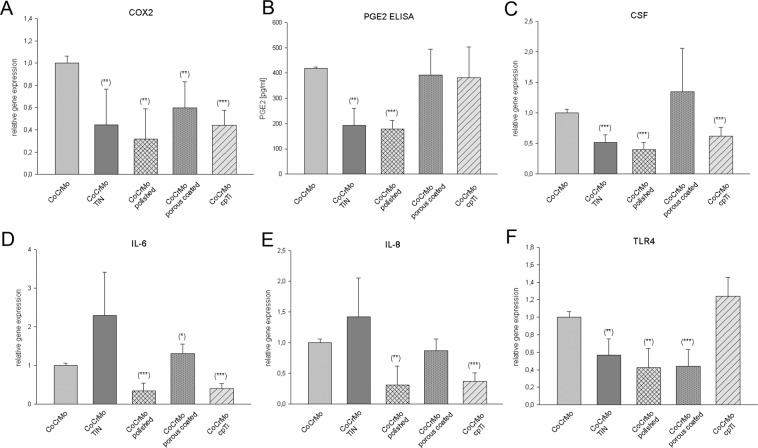
Effects of CoCrMo surface modifications on the expression of inflammatory markers and PGE2 release. (**A**) Compared to the CoCrMo control group all surface modifications led to significantly reduced relative COX2 gene expression. (**B,C**) Also, PGE2 production and CSF expression were significantly reduced on TiN and polished surfaces. (**D,E**) IL-6 and IL-8 expression were significantly decreased in polished, porous coated, and cpTi surfaces. (**F**) TiN, polished, and porous coated affected the expression of TLR4 (mean ± SD; N = 6; measured in triplicates).

Inflammation is closely related to bone metabolism. The link between altered bone metabolism and inflammation is not limited to systematic inflammatory diseases^16^. To investigate other pro-inflammatory effects, we measured both the relative mRNA expression of cyclooxygenease 2 (COX2) and corresponding prostaglandin E2 (PGE2) production. All surface modifications diminished the COX2 expression significantly in relation to the original CoCrMo alloy (Fig. 5A). The polished surface in particular reduced COX2 expression by nearly two thirds. Likewise, the PGE2 production was significantly reduced on this surface from 418 ± 4.6 pg/ml (CoCrMo) to 192 ± 55.43 pg/ml, **p = 0.0037 (TiN), 179 ± 26.56 pg/ml, ***p = 0.00027 (polished), 393 ± 83.14 pg/ml (porous coated), and 382 ± 99.31 pg/ml (Fig. 5B). The key inflammatory cytokines CSF, interleukin 6 (IL-6) and IL-8 showed highly significant reductions on the polished surface and the cpTi coating (Fig. 5D,E). All corresponding values are presented in Table 2.

**Table 2:** The expression levels of the inflammatory markers were normalized to the reference genes and the relative expression ratio was expressed as 2^ΔΔCt^. CoCrMo control cells served as the reference value (ratio = 1). All values were mean ± SD of independent experiments performed in triplicate (N = 6).

	COX2	CSF	IL-6	IL-8
TiN	0.45 ± 0.31 **p = 0.0033	0.51 ± 0.12 ***p = 0.0002	2.29 ± 1.12 n.s.	1.42 ± 0.63 n.s.
polished	0.32 ± 0.26 **p = 0.0012	0.40 ± 0.12 ***p = 6.5E-5	0.35 ± 0.19 ***p = 0.0002	0.31 ± 0.19 **p = 0.006
porous coated	0.59 ± 0.23 **p = 0.0015	1.43 ± 0.71 n.s.	1.31 ± 0.25 *p = 0.03	0.87 ± 0.19 *p = 0.03
cpTi	0.44 ± 0.14 ***p = 3.8E-8	0.81 ± 0.01 ***p = 6.7E-5	0.41 ± 0.12 ***p = 1.08E-5	0.37 ± 0.14 ***p = 2.01E-5

The innate immune response contributes to the development and maintenance of inflammation. Inflammation is tightly regulated by the toll-like receptor (TLR) family. TLR4 is expressed in the muscoskeletal system, where it plays a key role in the regulation of the inflammatory environment^17^. Compared to the original CoCrMo alloy, relative TLR4 expression was significantly reduced in TiN surfaces (0.56 ± 0.19, **p = 0.0049), polished (0.42 ± 0.21, **p = 0.003), and porous coated (0.44 ± 0.19, ***p = 2.92E-5) surfaces (Fig. 5F).

## Discussion

Developing new medical implants and improving their performance requires a solid understanding of biocompatibility, in particular how the host responds to biomaterials. A central question in this regard is whether biomaterials release degradation products. If so, the toxicity, immunogenicity and mutagenicity of these products must be determined, taking into account the concentrations at which they are released. Research currently focuses on altering the surface of the material in a way that leads to cell attraction, as this would create an ideal bond between the biomaterial and the tissue it is embedded in. An example of this approach is to make the surface more rough and create a more complicated topography. The effects of micro- and nanoscale topography on osteoblasts have been investigated in numerous studies^18–20^. The majority of these studies reached the conclusion that the proliferation of bone cells is best aided by nanoscale topography. This is because irregular shapes on the biomaterial’s surface interact with adsorbed cell-adhesion molecules, which leads to better adhesion of osteoblasts. As a result, proliferation of osteoblasts is enhanced^19–21^.

In our study, we investigated the biocompatibility of different CoCrMo alloy surface modifications: a porous coated surface, a polished surface, titanium nitride (TiN) coating, and a coating with commercially pure titanium (cpTi); we studied the adhesion potential of human osteoblasts and the expression of inflammatory markers. All materials were provided by a commercial manufacturer of orthopedic implants. Our biocompatibility studies showed very good compatibility for the TiN hard coatings. The TiN coating prevents the base material from coming into contact with the tissue in the vicinity, as well as stopping particles and ions from being released due to corrosion and abrasion. Due to the strong reduction in the release of potentially allergenic metal ions from the alloys, the TiN coating is particularly suitable for allericy patients who are highly sensitized for cobalt, chromium or nickel^22,23^. Likewise, the porous coated surface increased the biocompatibility significantly, compared to the untreated CoCrMo alloy. Fortunately, none of the investigated materials were cytotoxic to osteoblasts. Tissue integration of an implant is directly correlated with cellular adhesion and a positive interaction between hard and soft tissue cells with the implant surface. Osteoblast migration, attachment and subsequent proliferation are heavily influenced by surface characteristics^24,25^. A multitude of cell adhesion molecules are produced by cells derived from the osteoblast lineage and these regulate interactions between cells and surfaces. This study aimed to measure expression of the extracellular matrix protein fibronectin, as well as various integrin receptor subunits, as these transmembrane receptors are involved in cell-cell and cell-matrix interactions. Finally, we also measured expression of vinculin, a membrane-cytoskeletal protein that is involved in linking integrins to the actin cytoskeleton.

Fibronectin expression was not changed by the surface modifications tested here. In contrast, integrin subunit expression was significantly reduced after culture on TiN and porous coated surfaces and interestingly, also on polished surfaces. In osteoprogenitors, the β1 integrin sub-family show the highest expression of integrins. β1 integrins are also the most important cell adhesion mediator in this cell type^26^. There is a lack of consensus on whether osteoblasts express α-subunits and integrin heterodimers. However, numerous studies conducted in osteoblasts and bone cultures have demonstrated expression of integrins α1β1, α2β1, α3β1, α5β1 and αvβ3 as well as their subunits^27,28^.

α-subunit expression in primary bone cultures was additionally confirmed by flow cytometry, immunocytochemistry, immunoprecipitation as well as Northern blot^26,27,29,30^. Our RT-PCR data revealed that TiN coating, polished, and porous coated surfaces decreased the expression of ITG- β1 and ITG- α1, as well as α3, and α5 subunits in human osteoblasts significantly.

In addition to the regulation of adhesion potential, we found that TiN, polished, and porous coated surface modifications in particular, stimulated an anti-inflammatory response by osteoblasts. Interestingly, despite their structural role, osteoblasts are also able to induce an inflammatory response^31,32^. COX2 expression decreased on all surface modifications, whereas the PGE2 and CSF expression was significantly reduced only with TiN and polished surfaces. The most significant reduction in all inflammation markers, including IL6 and IL8, was observed on the polished surface. Toll-like receptor (TLR) pathways may be involved in the inflammatory response of osteocytes to biomaterials. TLRs sense foreign bodies such as lipopolysaccharide and are also involved in the inflammation cascade^33^. In this context, TLR4 plays an essential role. This fact is supported by our data, which showed that expression of TLR4 was also significantly decreased.

As can be clearly seen from the data, the different coatings differ considerably in their effect on the cell biology of osteoblasts. The ceramic TiN and the porous coated surfaces are especially notable for their high biocompatibility. In general, all modified surfaces showed improvements compared to the uncoated CoCrMo alloy. With regard to adhesion properties and expression of inflammation markers, TiN and polished surfaces showed particularly positive effects.

## Methods

### CoCrMo alloy surface modifications

The CoCrMo discs were manufactured by Implantcast (Buxtehude, Germany) using a precision casting process, the special coating was produced by DOT Ltd (Rostock, Germany). According to ISO 5832-4 specification, the alloy contains 59–65% Co, 26.8–30% Cr, 4.5–7% Mo, and less than 1% Ni, Fe, C, Si, and Mn. For the CoCrMo porous coated surface, three layers of 250–355 µm sized balls were applied to the alloy using sintering, resulting in a coating thickness of 700–1060 µm and a porosity of 30–40%. The tensile strength was >34.5 MPa (pull off test), and the shear strength was > 20 MPa (shear test). The ceramic surface coating with titanium nitride (TiN) is thought to be wear-reducing, anti-allergic, and more biocompatible. TiN coating is an additive process in which the coating is securely anchored in the implant surface. In this process, titanium atoms are released by electrical energy from a solid target, ionized and accelerated to the implant surface. There they combine with nitrogen molecules to TiN. Using physical vapour deposition, a 5.5 ± 1.5 µm thick golden yellow ceramic TiN layer with a mean surface roughness (R_a_) < 0.05 µm. The most important parameter for the quality of an applied layer is its adhesion to the substrate. In order to assess the adhesion of the TiN coating reliably, various qualitative tests are carried out on the layers. These tests include the Rockwell HRC indentation test according to VDI guideline 3824 and the mandrel bending test (HF 1–4). An adhesive coating shows only slight crack formation at the deformed edge and no layer spalling. For the TiN layer, a very good adhesive strength could also be determined in the mandrel bending test ( >22 MPa).

The coating with commercially pure titanium (cpTi) using vacuum plasma spraying resulted in a rough and porous surface layer with a thickness of 250–350 µm and a porosity of 20–40%. Mean surface roughness was determined to be (R_a_) 50 ± 15 µm, with a tensile strength of >22 MPa, and a shear strength below >20 MPa. All materials were manufactured as discs with a diameter of 14 mm and a thickness of 1 mm, in order to match the 24 well plates used in cell culture experiments. Gamma irradiation sterilization was performed according to standardized protocols.

### Scanning electron microscopy (SEM)

For the SEM investigations a FEI Quanta 250 FEG (Thermo Fisher Scientific, Hillsboro, OR) was used under high vacuum conditions and 20 kV high tension. The micrographs were recorded with the Everhart-Thornley-Detector in secondary electron (SE) mode. The fracture surface was sputtercoated with a 10 nm thin layer of gold in order to provide sufficient electrical conductivity. The conditions used in the energy-dispersive X-ray spectroscopy (EDX) experiments were as follows: 60 s, 20 kV high tension and a Spotsize of 4.5 with a 30 mm² Octane Elect Plus Silicon Drift Detector by EDAX Ametek, (NJ, USA) and APEX Standard Software V1.3.1 from 6^th^ July 2019.

### Cell culture

hFOB1.19 osteoblasts (Homo sapiens, CRL-11372TM, ATCC, Manassas, VA) were cultured in DMEM/F12 (GIBCO, Invitrogen, Darmstadt, Germany) with 10% fetal bovine serum (FBS), 1% L-glutamine, 100 units/ml penicillin and 100 μg/ml streptomycin (all GIBCO, Invitrogen, Darmstadt, Germany). Incubation was performed at 34 °C in a humidified atmosphere with 5% CO_2_. Cell culture media was changed every 3 days under aseptic conditions.

### Viability assay

Osteoblasts were seeded on CoCrMo discs inserted in 24-well Corning Costar Ultra-Low attachment multiwell plates (Corning Inc., Corning, NY). Cells were seeded at a density of 2.5 × 10^4^ hFOB cells per well. The CellTiter-Glo Luminescence Cell Viability Assay (Promega, Madison, MA) was carried out after 4 days of incubation, according to the manufacturer’s instructions. Background reference values were derived from the culture media. Absorbance values were measured with the Lumistar microplate luminometer (BMC Labtech, Ortenberg, Germany).

### Cytoxicity assay

The activity of Lactate Dehydrogenase (LDH) was measured with the CytoTox-ONE Homogeneous Membrane Integrity Assay (Promega, Madison, MA). Following four days of incubation, supernatants of 2.5 × 10^4^ hFOB cells were collected to measure cell damage. 50 µl of working solution were mixed with 50 µl of supernatant in white 96-well microtiter plates, then incubated in the dark for 10 minutes at room temperature. The reaction was terminated by adding 50 µl of stop solution. Fluorescence was measured at 560/590 nm using a Fluostar (BMC Labtech, Ortenberg, Germany).

### Enzyme immuno assay ELISA

Ready-to-use Sandwich ELISA kits for detection of human Prostaglandin E2 (PGE2; ENZO, Farmingdale, NY) and Fibronectin (Abcam, Cambridge, UK) were utilized. After four days of incubation, undiluted supernatants were treated according to the supplied protocol. Each measurement was performed in duplicate at 450 nm using a SPECTROstar microplate reader (BMC Labtech, Ortenberg, Germany).

### Real-time RT-PCR

Total RNA was isolated from osteoblasts cultured on the various CoCrMo alloy surfaces via the RNeasy Mini Kit (including DNase-I treatment) according to the manufacturer’s protocol (Qiagen, Hilden, Germany). One μg RNA was reverse transcribed using the iScript cDNA Synthesis Kit, (BioRad Laboratories Inc., Hercules, CA). A mix of oligo(dT) and random hexamer primers was employed. SsoAdvanced Universal SYBR Green Supermix (Bio-Rad) was used for amplification. Amplification was measured in a CFX96 Touch (BioRad). All qPCR experiments consisted of a standard 3-step PCR temperature protocol (annealing temperature of 60 °C). A melting-curve step was included at the end to confirm a single gene-specific peak and detect primer dimerization. Relative quantification of expression levels was determined by the ΔΔCt method. The geometric mean of the internal controls TBP (TATA-box binding protein) and RPLP0 (ribosomal protein, lateral stalk, subunit P0), was used. The expression levels (Ct) of target genes were normalized to the reference genes (ΔCt). The difference between the test sample ΔCt and the control sample ΔCt resulted in the ΔΔCt value. The expression ratio was then expressed as 2^ΔΔCt^. QuantiTect primer assays (Qiagen) were used and are listed here: vinculin, TNFα, IL-6, ITG-β1, ITG-α1, ITG-α3, ITG-α5, COX-2, MMP13, TLR4, and M-CSF-1.

### Statistical analysis

Differences between groups were measured using Student’s unpaired t-test and the exact Wilcoxon test with PASW statistics 18 software (IBM Corporation, Somers, NY). Two-sided P-values (*p* < 0.001***; *p* < 0.01**; *p* < 0.05*) were considered to be statistically significant. SigmaPlot 14.0 (Systat Software Inc., San Jose, CA) was used to produce figures.
